# Parental Hesitancy toward Seasonal Influenza Vaccination for Children under the Age of 18 Years and Its Determinants in the Post-Pandemic Era: A Cross-Sectional Survey among 1175 Parents in China

**DOI:** 10.3390/vaccines12090988

**Published:** 2024-08-29

**Authors:** He Cao, Siyu Chen, Yijie Liu, Kechun Zhang, Yuan Fang, Hongbiao Chen, Tian Hu, Rulian Zhong, Xiaofeng Zhou, Zixin Wang

**Affiliations:** 1 Longhua District Center for Disease Control and Prevention, Shenzhen 518110, China; caohe0312@163.com (H.C.); zkc1317@yeah.net (K.Z.); gesila2021@163.com (H.C.); ht1137571641@126.com (T.H.); zrllan23893526@sina.com (R.Z.); zxf20220312@163.com (X.Z.); 2 Jockey Club School of Public Health and Primary Care, Faculty of Medicine, The Chinese University of Hong Kong, Hong Kong, China; chensiyu@link.cuhk.edu.hk (S.C.); 1155201122@link.cuhk.edu.hk (Y.L.); 3 Department of Health and Physical Education, The Education University of Hong Kong, Hong Kong, China; lunajoef@gmail.com

**Keywords:** seasonal influenza vaccination, vaccine hesitancy, children, parents, social media, China

## Abstract

Children’s susceptibility to influenza increased after COVID-19 control measures were lifted. This study investigated parental hesitancy toward seasonal influenza vaccination (SIV) for children and its determinants in the post-pandemic era. An online survey of full-time adult factory workers was conducted in Shenzhen, China in December 2023. This analysis was based on 1175 parents who had at least one child under the age of 18 years. Among all parents, 37.1% were hesitant to have their index child receive SIV. Mothers exhibited lower parental hesitancy toward SIV compared to fathers (31.9% versus 41.3%, *p* < 0.001). After adjusting for significant background characteristics, mothers and fathers who were more satisfied with the SIV health promotion materials, perceived more severe consequences of seasonal influenza for their children, and perceived more benefits, cues to action, and self-efficacy related to their children’s SIV were less likely to exhibit hesitancy toward SIV. Higher frequency of exposure to information about the increasing number of patients or severe cases due to seasonal influenza and other upper respiratory infections on social media was associated with lower parental hesitancy toward SIV among fathers but not mothers. There is a strong need to address parental hesitancy toward SIV for children in the post-pandemic era.

## 1. Introduction

Children, especially young children, have a high hospital admission rate due to influenza infection, which increases healthcare burdens and healthcare system workload across countries [1,2]. In China, an average of 88,000 excess deaths are attributed to seasonal influenza annually [3]. The China national surveillance data reported 3,512,000 influenza-like illness (ILI) between 2022 and 2023; approximately 8–20% of these ILI cases were children under the age of 18 years [4]. During the flu season, the incidence rate of influenza among children usually falls between 20% and 30% in China; such an incidence rate can reach 50% in some flue seasons [5,6]. The mortality rate of seasonal influenza among children in China ranged from 0.37 to 0.91 per 100,000 children over the past two decades [7]. Mathematical models estimated a 10–60% increase in the population susceptibility to influenza after COVID-19 control measures were lifted, which might lead to a large increase in the peak magnitude and epidemic size of seasonal influenza in the post-pandemic era [8]. During the 2022–2023 flu season, there was a large increase in the number of cases of influenza-like illness and confirmed cases of influenza in China, greatly exceeding the figures observed during and before the time of COVID-19 [9].

Seasonal influenza vaccination (SIV) is an important measure to protect children and the wider population [10]. Previous studies demonstrated that SIV could significantly reduce the risk of life-threatening influenza illness and hospitalization due to seasonal influenza infection by 59–75% and 52–61%, respectively [11,12]. Therefore, the World Health Organization (WHO) considers children aged 6–59 months as a priority group to receive SIV [13]. Currently, over 60% of all countries include SIV in their immunization plans for children [14]. Health authorities in China have recommend annual SIV for children aged 6 months or older since 2014 [15,16,17]. The main scheme of the national SIV program in China offers a self-financed inactivated trivalent vaccine (TIV) at CNY 45 (USD 6.2) per dose and a quadrivalent (QIV) vaccine at CNY 153 (USD 22) per dose for children [15,18]. A pilot scheme under the national program, which provided free TIV to children aged between 7 and 17 years, was launched in Shenzhen and some other Chinese cities in 2022 [18,19]. If parents or caregivers prefer QIV for their children, they need to follow the arrangement of the main scheme. However, SIV coverage remained inadequate among children in China. The overall SIV coverage among children in China was 13–40.7% before the national SIV program was implemented (2006–2014) and was 11.9–35.7% after the rollout of the national SIV program in 2014 [20,21,22].

Parents are the main decision makers for their children’s vaccination. Vaccine hesitancy is defined as an attitude that oscillates between acceptance and refusal of vaccination despite the availability of a vaccine against a particular disease [23]. If parents or caregivers were unwilling to receive a SIV for themselves, they might be reluctant to have their children receive the same vaccination [24]. Three studies were identified through our literature review that investigated parental hesitancy toward SIV for their children in China, which revealed a relatively high level of hesitancy, ranging from 34.1% to 43.2% [25,26,27]. Determinants considered by these studies were limited to background characteristics of the parents or their children (e.g., age of the children, socioeconomic status, history of seasonal influenza infection), and parents’ attitudes toward SIV (i.e., perceived benefits of SIV, concerns about protection conferred by SIV, and recommendations given by family members related to SIV) [25,26,27]. However, all these studies were conducted during or before the time of COVID-19.

The socio-ecological model suggests that determinants of a health behavior were at five different levels, including the individual, interpersonal, organizational, environmental, and public policy levels [28]. Such a model was commonly used to investigate determinants of vaccination uptake [29,30,31,32,33]. This study considered determinants at the first two levels. At the individual level, in addition to perceptions related to SIV, this study considered parents’ satisfaction with SIV health promotion materials and children’s use of personal preventive measures. Health authorities in China produce numerous materials promoting SIV. It is unclear whether these materials have fully addressed parents’ concerns related to SIV or provide a sufficient amount of information to facilitate parents to make a decision whether to have their children receive SIV [34,35]. Studies conducted among older adults in China showed that higher level of satisfaction with vaccination promotion materials produced by the government were associated with lower hesitancy to receive COVID-19 vaccination [36,37]. Based on the lessons learned during the COVID-19 pandemic, it is common for parents in China to ask their children to wear facemasks in public spaces to prevent respiratory infections. It is possible that parents would think facemask wearing is sufficient to protect their children against seasonal influenza, and hence they have no need to have such children receive SIV. Moreover, while motivations for wearing facemasks are important, the frequency of use is equally crucial in mitigating pathogen spread [38]. Regular and consistent use during potential exposure situations, such as in crowded environments or during respiratory illness seasons, may play a critical role in mitigating the spread of pathogens. Therefore, comprehending both the motivations behind and the practical application of protective measures, such as mask-wearing and SIV, is essential for fully understanding their impact on public health outcome. To our knowledge, no study has tested such a hypothesis.

At the interpersonal level, social media platforms are important sources for obtaining health-related information across countries [39]. Infectious disease outbreaks, including seasonal influenza, are usually hot topics on social media platforms [40]. Previous studies highlighted the impacts of information exposure on vaccination uptake. Chinese people who had higher frequency of exposure to anti-vaccine content on social media were less likely to receive SIV [41]. In contrast, Chinese parents who had a higher frequency of exposure to information that was supportive of COVID-19 vaccination were more likely to have their children receive such vaccination [42]. In addition, exposure to misinformation related to vaccination on social media may increase vaccine hesitancy [43]. People who often consider the veracity of the information on social media were less likely to be affected by such misinformation [44,45]. Previous studies showed that higher frequency of thoughtful consideration was associated with higher vaccination uptake [45,46]. These variables at the interpersonal level were also considered by this study. 

To address the knowledge gaps, this study investigated parental hesitancy toward SIV for children and its determinants in the post-pandemic era. In this study, we focus on a subgroup of parents who were full-time factory workers in Shenzhen, China. The population size of factory workers is large in Shenzhen. Of the 13 million residents, 34.3% were factory workers [47]. Most of the factory workers were internal migrants with low education level and socioeconomic status and poor living conditions [48]. Previous studies highlighted the influence of socioeconomic status on the vulnerability to infectious diseases and access to vaccination services [49]. Understanding parental hesitancy toward SIV and its determinants among factory workers may be useful to address potential health inequality. Potential determinants considered by this study included those at the individual level (satisfaction with SIV health promotion materials produced by the government, children’s use of facemasks, and perceptions related to SIV) and the interpersonal level (frequency of exposure to information on social media and thoughtful consideration of the veracity of such information). Our hypothesis was that parental hesitancy toward SIV for their children would be influenced by factors at both the individual and interpersonal levels.

## 2. Materials and Methods

### 2.1. Study Design

This was a secondary analysis of a cross-sectional survey investigating SIV uptake and attitudes among adult factory workers in Shenzhen, China conducted between 7 December 2023 and 21 December 2023. The analysis was based on a sub-sample of factory workers who had at least one child under the age of 18 at the time of the survey. Shenzhen, bordering Hong Kong to the north, is a special economic zone and one of the most developed cities in China. Factories in Shenzhen are concentrated in its Longhua district. In 2020, there were more than 1500 factories with one million workers in the Longhua district of Shenzhen [50].

### 2.2. Participants and Data Collection

The inclusion criteria of the original survey were as follows: (1) full-time employees of factories in Shenzhen, (2) aged 18 years or above, (3) able to read simplified Chinese, and (4) have a smartphone with internet access. In Shenzhen, all employees of factories are required to receive an annual physical examination to renew their permits to work in the factories. Six organizations, including five hospitals (three public and two private) and the District Center for Disease Control and Prevention (CDC), have been providing such physical examinations in the Longhua district of Shenzhen. During the study period, trained fieldworkers screened all adults receiving physical examinations at these six organizations and invited them to join this study. The fieldworkers emphasized that participation was voluntary and anonymous, and refusal would have no consequences. The fieldworkers also explained data safety to participants (i.e., data would be kept confidential and only used for research purposes). A similar sampling strategy was used in a number of studies to obtain a more representative sample of factory workers [51,52]. 

A user-friendly online survey platform, the Questionnaire Star, was used to implement the survey. Participants could access the questionnaire by scanning a quick response (QR) code using their mobile devices. Participants completed an electronic consent form before they could start answering the online survey. The survey platform allowed each mobile device to access the online survey once in order to avoid duplication. Participants were asked not to disseminate the QR code to others. The survey platform confirmed that all required questions were completed before participants could submit their questionnaires. Participants could review and make changes to their responses before submission. A CNY 10 electronic cash coupon (USD 1.5) was given to each participant once he/she completed the online survey which took about 20 min to finish. The data, which were stored in the survey platform server, were encrypted and password protected. Only the corresponding author was allowed to access the database. We extracted a sub-sample of participants having ≥1 child aged under 18 years for this study. Ethical approval for this study was obtained by the Longhua District CDC (reference: 2020001).

### 2.3. Sample Size Planning

Similar to previous surveys targeting factory workers in Longhua, the target sample size of the original study was 2000 [51,52]. We expected the sample size for this secondary analysis to be around 800 (about 40% of the participants had a child under the age of 18 years). If the level of parental hesitancy toward SIV was 10–50% in parents with a facilitating condition for SIV, a sample size of 800 could detect the smallest odds ratio of 1.29 between parents with and without a facilitating condition with power = 0.80; alpha = 0.05. The sample size calculation was performed using PASS 11.0 (NCSS, LLC., Kaysville, UT, USA).

### 2.4. Measurements

#### 2.4.1. Development of the Questionnaire

Epidemiologists, health psychologists, clinicians, and CDC staff formed a panel to develop the questionnaire for this study. Ten factory workers were purposively recruited to test the questionnaire. All these workers indicated that the contents of the questionnaire were easy to understand and that the length was acceptable. The panel then finalized the questionnaire based on the feedback. Data from these ten factory workers were not included in the analysis. 

#### 2.4.2. Background Characteristics

The questionnaire collected information on sociodemographic characteristics (i.e., age, sex assigned at birth, ethnicity, permanent residency in Shenzhen, relationship status, education level, monthly personal income, and whether they were frontline workers or management staff). Participants also reported a history of confirmed SARS-CoV-2 infection, COVID-19 vaccination status, and whether they had confirmed seasonal influenza and other upper respiratory infections in the past six months. In the case that participants had more than one child under the age of 18 years, they referred to the one whose birthday was closest to the survey date (the index child) when answering the following questions: (1) age of the index child, (2) whether the index child had a history of confirmed SARS-CoV-2 infection, and (3) whether the index child was confirmed to have seasonal influenza or other upper respiratory infections in the past six months. 

#### 2.4.3. SIV Uptake and Hesitancy

Parents reported whether their index children had received SIV for the incoming flu season. If parents answered “no”, the questionnaire asked the parents to rate their likelihood of having their index children receive a SIV in the next year from 1 = very unlikely, 2 = unlikely, 3 = neutral, 4 = likely, to 5 = very likely. We followed the definition in published studies and defined vaccine hesitancy as “very unlikely”, “unlikely”, or “neutral” [53,54]. We used two similar questions to measure participants’ hesitancy to receive SIV for themselves.

#### 2.4.4. Variables at the Individual Level

We adapted a scale validated in the Chinese population to measure level of satisfaction with SIV health promotion materials produced by the government (i.e., advertisements, posters, and others) for this study [45]. The phrase “COVID-19 vaccination” in the original scale was replaced by “SIV” in this study [45]. In the scale, three items measured whether these health promotion materials (1) could offer appropriate amount of information related to SIV, (2) could address their concerns related to SIV, and (3) were helpful for them to make a decision about whether to receive SIV (response categories: 1 = disagree, 2 = neutral, and 3 = agree). The Cronbach alpha of the Satisfaction with Health Promotion Materials was 0.76 in this study. 

Two items measured the index children’s use of personal preventive measures, including frequency of wearing facemasks on public transportation and at schools in the past month (response categories: 1 = never/not applicable, 2 = sometimes, 3 = always, and 4 = every time). 

Three scales were constructed based on the Health Belief Model (HBM), a behavioral change theory commonly used to inform vaccination promotion [55]. They were the following: (1) the 3-item Perceived Severity Scale (e.g., “The index child will have severe symptoms due to seasonal influenza infection”), (2) the 2-item Perceived Benefit Scale (e.g., “SIV is highly effective in protecting the index child against seasonal influenza”), and (3) the 3-item Perceived Barrier Scale (e.g., “The index child will have severe side effects after receiving SIV”). The Cronbach’s alphas of these scales were acceptable, ranging from 0.67 to 0.78. In addition, we adapted three single items validated in the Chinese population to measure the index child’s susceptibility to seasonal influenza (“The index child has a high chance of contracting seasonal influenza”), cue to action (“Your significant others would suggest you to vaccinate the index child against seasonal influenza”), and perceived self-efficacy (“It is easy for you to vaccinate the index child against seasonal influenza if you want to”) [45]. The response categories to the aforementioned scale items were 1 = disagree, 2 = neutral, and 3 = agree. 

#### 2.4.5. Independent Variables at the Interpersonal Level

Four items were constructed for this study to measure frequency of information exposure related to seasonal influenza or SIV on common social media platforms in China (e.g., WeChat moments, Weibo, TikTok, and Red) in the past month. This information included parents’ testimonials about their children’s severe symptoms following seasonal influenza or other upper respiratory infections, information regarding difficulties in seeking medical treatments for seasonal influenza and other upper respiratory infections for children, and information about an increasing number of patients in the hospitals or severe cases due to seasonal influenza and other upper respiratory infections (response categories: 1 = almost none, 2 = seldom, 3 = sometimes, and 4 = always). We adapted a validated item to measure thoughtful consideration about the veracity of information specific to SIV in the past month [44]. 

### 2.5. Statistical Analysis

Descriptive statistics of all studied variables were presented. The dependent variable was parental hesitancy toward SIV for the index child. The associations between each of the background characteristics and the dependent variable were assessed using univariate logistic regression models. Multivariate logistic regression models were then fitted to analyze the associations between each independent variable of interest and the dependent variable after adjusting for all background characteristics with *p* < 0.05 in the univariate analysis. We performed subgroups analysis among mothers and fathers, as mothers in China usually are the primary decision makers for children’s vaccination [56]. We reported crude odds ratios (ORs), adjusted odds ratios (AORs), and their 95% confidence intervals (CIs) in the results. We assessed multicollinearities using the variance inflation factor (VIF). When a variable exceeded a VIF value of 5, it would be adjusted or eliminated if theoretically redundant. We assessed the model fits of the logistic regression models using Hosmer and Lemeshow goodness-of-fit tests. An insignificant Hosmer and Lemeshow goodness-of-fit test result (*p* > 0.05) indicates that there is no significant evidence of a systematic lack of fit in the model [57,58]. In addition, correlations between information exposure on social media and perceived susceptibility and severity of seasonal influenza were analyzed using logistic regression models. The analyses were performed using SPSS (version 29.0; IBM, Armonk, NY, USA). A significance level of *p* < 0.05 was used.

## 3. Results

### 3.1. Background Characteristics

In the original survey, 3122 eligible factory workers were approached by the fieldworkers, of whom 2653 participants completed the online survey. A total of 1175 participants with ≥1 child under the age of 18 years were included in our analysis. Among the parents, 54.4% were over 30 years old, 55.2% were fathers of the index child, and 54.2% had a monthly personal income of less than CNY 5000 (USD 694) (54.2%). The majority of the parents had attained tertiary education (63.4%), worked as frontline workers (66.6%), had a history of confirmed SARS-CoV-2 infection (84.6%), and received at least three doses of COVID-19 vaccination (64.3%). About one-quarter of them self-reported having confirmed seasonal influenza (28.4%) or other confirmed upper respiratory infections (22.2%) in the past six months. Over half of the index children were over 6 years old (58.8%) and had a history of confirmed SARS-CoV-2 infection (72.4%). As reported by the parents, 27.6% and 30.8% of the index children had confirmed seasonal influenza and other upper respiratory infections in the past six months, respectively (Table 1). The differences in background characteristics between subgroups of female and male participants who had at least one child are presented in Table 1. 

### 3.2. SIV Uptake and Hesitancy

The SIV uptake rate for the incoming flu season was 34.0% among the index children. Among 776 participants whose index child had not received SIV, 43.8% intended to have such children receive SIV in the next year. Among all parents, 37.1% were hesitant to have their index children receive SIV, and 48.2% were hesitant to receive SIV themselves (Table 2). As compared to fathers, mothers had a lower level of parental hesitancy toward SIV for their children (41.3% versus 31.9%; *p* < 0.001) (Table 2).

### 3.3. Independent Variables of Interest at the Individual and Interpersonal Levels

About half of the parents agreed that the health promotion materials produced by the government could offer the appropriate amount of information related to SIV (52.9%) and address their concerns related to SIV (48.9%) and were helpful for them in deciding whether to receive SIV (51.1%). The mean and standard deviation (SD) for scores of scales and items measuring perceptions of SIV are presented in Table 2. Among the index children, 32.1% and 25.5% wore facemasks every time when on public transportation and at schools in the past month, respectively (Table 2).

In the past month, over half of participants were sometimes/always exposed to the following information on social media platforms: testimonials given by parents about their children’s severe symptoms following seasonal influenza or other upper respiratory infection (57.3%), difficulties in seeking medical treatments for seasonal influenza and other upper respiratory infections for children (58.3%), increasing number of patients in the hospitals (64.3%), or severe cases (60.1%) due to seasonal influenza and other upper respiratory infections. Over 60.0% of the parents sometimes/always considered the veracity of information specific to SIV in the past month (Table 2).

Higher frequency of exposure to the aforementioned information on social media platforms was significantly correlated with perceived higher susceptibility and higher severity of seasonal influenza for the index child (all *p* < 0.001) (Table A1). 

As compared to fathers, mothers perceived more severe consequences of seasonal influenza infection for their children (*p* < 0.001) and perceived more benefits (*p* < 0.001), cues to action (*p* = 0.003), and self-efficacy related to SIV for their children (*p* < 0.001). In addition, mothers had a higher frequency of exposure to information related to the difficulties in seeking medical treatments for seasonal influenza for children (*p* = 0.001) and increasing number of patients (*p* < 0.001) or severe cases (*p* = 0.007) due to influenza or other upper respiratory infections on social media platforms than fathers. Moreover, mothers were more likely to consider the veracity of information specific to SIV than fathers (*p* = 0.03) (Table 2). 

### 3.4. Factors Associated with Parental Hesitancy toward SIV for the Index Child

Participants who were older and were management staff, had a higher level of education, were mothers of the index children, or self-reported having confirmed seasonal influenza or other upper respiratory infections were less likely to have parental hesitancy toward SIV for the index children. In addition, a history of confirmed SARS-CoV-2, seasonal influenza, and other upper respiratory infections among the index children was associated with lower likelihood of parental hesitancy toward SIV (Table 3). The relationships between background characteristics and the dependent variables varied among mothers and fathers as shown in Table 3. 

After adjusting for significant background characteristics, parents who were more satisfied with the governmental SIV health promotion materials (AOR: 0.75, 95%CI: 0.69, 0.80), perceived a higher risk (AOR: 0.85, 95%CI: 0.73, 0.99) and more severe consequences (AOR: 0.83, 95%CI: 0.77, 0.88) of seasonal influenza for their index children, perceived more benefits (AOR: 0.67, 95%CI: 0.60, 0.73), barriers (AOR: 0.93, 95%CI: 0.87, 0.99), and self-efficacy related to their index child’s SIV (AOR: 0.59, 95%CI: 0.50, 0.70), and received more suggestion from their significant others to vaccinate their index child against seasonal influenza (AOR: 0.59, 95%CI: 0.50, 0.69) were less likely to have parental hesitancy toward SIV. Higher frequency of exposure to testimonials given by parents about their children’s severe symptoms following seasonal influenza or other upper respiratory infections (AOR: 0.85, 95%CI: 0.76, 0.96), difficulties in seeking medical treatments for seasonal influenza and other upper respiratory infections for children (AOR: 0.87, 95%CI: 0.77, 0.99), or the increasing number of patients in the hospitals or severe cases due to seasonal influenza (AOR: 0.85, 95%CI: 0.75, 0.96) and other upper respiratory infections (AOR: 0.88, 95%CI: 0.77, 0.99) was also associated with lower likelihood of having parental hesitancy toward SIV. In addition, parents’ hesitancy to receive SIV for themselves was associated with higher parental hesitancy toward SIV (AOR: 4.46, 95%CI: 3.38, 5.90) (Table 4). VIF values for these logistic regression models ranged from 1.36 to 2.21. Therefore, adjustment was unnecessary for the variables. There is no significant evidence of a systematic lack of fit in the aforementioned logistic regression models (Hosmer and Lemeshow test ranged from 0.09 to 0.87). 

Determinants of parental hesitancy toward SIV for children were similar between mothers and fathers, with the exception of exposure to some information on social media platforms. Frequency of exposure to information related to increasing number of patients or severe cases due to influenza or other upper respiratory infections was associated with lower parental hesitancy toward SIV among fathers, but not among mothers. There is no significant evidence of a systematic lack of fit in the logistic regression models in subgroup analysis (Table 4).

## 4. Discussion

This is one of the first studies to investigate parental hesitancy toward SIV for their children in the post-pandemic era. The strengths of this study include its high relevance to population-level responses to potential seasonal influenza outbreaks in the post-pandemic era, considering determinants at both the individual and interpersonal levels, and having a relatively large sample size with a high response rate. Our findings provide useful data to predict SIV coverage among children in Shenzhen, China, and expand the application of the socio-ecological model in explaining health-related behaviors. This study can inform evidence-based service planning and health promotion of SIV for children in China in the post-pandemic era. 

Only 34.0% of children received SIV for the incoming flu season. Such uptake rate was lower than the figures reported in some developed countries (e.g., 56% in England and 55% in the United States) [59,60] and was much lower than the WHO’s recommended SIV coverage for children (75%) [61]. Among the parents whose children had not received SIV, less than half of them intended to vaccinate their children against seasonal influenza in the next year. The level of parental hesitancy toward SIV for children under the age of 18 years in this study (37.1%) was similar to those reported in China before and during the COVID-19 period (34.1–43.2%) [25,26,27] and was higher than those observed in the United States (19.5–25.8%) [62] and Italy (13.0%) [63]. There is large room for and a strong need to address parental hesitancy toward SIV for their children in China. 

Future health promotion should target mothers, as they are the primary decision makers regarding children’s vaccination in China and had lower level of parental hesitancy toward SIV compared to fathers [56]. In line with previous studies, older parents were less likely to have parental hesitancy toward SIV for their children. Younger parents may have less experience vaccinating themselves and their children as compared to their older counterparts [64]. Previous studies also suggested that younger parents perceived a low risk of severe consequences of seasonal influenza in children [65,66]. Therefore, older parents may be more receptive toward SIV for children. Higher income and a management staff occupation were associated with a lower likelihood of having parental hesitancy for vaccination of their children. Previous studies suggested that parents with better socio-economic status had higher health literacy, and hence were more aware of the importance of SIV [67,68]. More attention should be given to parents of low socio-economic status in future SIV promotion campaigns. Parents and/or their index children with confirmed seasonal influenza or other upper respiratory infections were less likely to have parental hesitancy toward SIV. It was possible that these parents perceived a greater threat of seasonal influenza and hence had higher motivation to vaccinate their children.

Our findings provided some empirical implications to inform evidence-based health promotion. Mothers’ and fathers’ hesitancy to receive SIV for themselves was associated with a higher level of parental SIV hesitancy for their children. Therefore, promoting SIV among parents may increase SIV coverage among both adults and children simultaneously. It is necessary to improve the current SIV health promotion materials produced by the government, as only half of the parents agreed that such materials could provide the appropriate amount of information, address their concerns, and facilitate their decisions related to SIV. Mothers and fathers who were more satisfied with the health promotion materials exhibited a lower level of parental hesitancy toward SIV. Most of the existing health promotional materials for SIV are created by scholars or healthcare professionals [69]. These materials seldom take into account the perspectives and needs of the end-users [70]. Co-creation, an approach involving collaboration among scholars, end-users, and other relevant stakeholders, was considered useful by previous studies to improve the health promotional materials [71,72]. This strategy has been shown to be useful to address complex issues and foster behavioral change [71]. In contrast to our hypothesis, children’s facemask wearing was not associated with parental hesitancy toward SIV. 

In line with previous findings, modifying perceptions based on the HBM might be useful strategies to reduce parental hesitancy toward SIV. In addition to the severe symptoms of seasonal influenza among children, both mothers and fathers are concerned about the negative impacts of seasonal influenza on their children’s academic performance and their healthcare burdens. It is potentially useful to highlight these consequences of seasonal influenza, as parents who perceived more severe consequences were less hesitant to vaccinate their children against seasonal influenza. Over 60% of parents perceived some benefits of SIV. Emphasizing SIV vaccine efficacy is useful as perceived benefits were associated with a lower likelihood of having parental hesitancy. Future programs should also consider involving significant others of parents to provide strong cues to action. Previous studies supported the feasibility of implementing school-based vaccination programs in China. Since 2007, the Department of Health and the Department of Education of Beijing, China have been collaborating to administer free SIV in primary and secondary schools [73]. In 2019, Shenzhen started to provide free SIV in primary and secondary schools [19]. In addition, school-based interventions were shown to be feasible and effective in improving SIV and HPV vaccination uptake among children in China [74,75]. These programs may effectively address parents’ time constraints for vaccinating their children, thereby enhancing parental self-efficacy regarding children’s SIV in Shenzhen. 

Seasonal influenza appeared to be a hot topic on social media, as about 60% of the parents were sometimes or always exposed to topics related to seasonal influenza on popular social media platforms in China in the past month. Our study suggested that information exposure on social media had differential impacts on parental hesitancy toward SIV between mothers and fathers. Higher frequency of exposure to statistics of influenza or other upper respiratory infections (e.g., number of patients and severe cases) were associated with lower level of parental hesitancy toward SIV among fathers, but not mothers. Studies suggested that males are more influenced by statistics when making a decision, as compared to females [76,77]. Future health promotion should be aware of such gender differences. 

This study had several limitations. First, failure to include altruistic factors was one major limitation of this study. SIV is an important measure to protect children and the wider population [10]. Altruistic motivation plays a significant role in shaping health behaviors. Individuals often engage in protective measures not only for personal benefits but also to protect others [78]. Previous studies showed that mask wearing behavior during the COVID-19 pandemic was mainly driven by the belief that collective mask usage could reduce community transmission risks and alleviate anxiety among people in Japan [79]. Altruistic motivation also plays a role in vaccination decisions, particularly in multi-generational households or those with an elderly member. Parents who prioritize the wellbeing of their elderly household members may have higher motivation to vaccinate their children [80]. Future studies should explore the impact of altruism and social norms on parental hesitancy toward SIV, which may improve public health strategies and lead to more effective interventions against infectious diseases. Second, we did not ask whether the participant was the primary caregiver of the child in this study. Third, all participants were factory workers. Although factory workers comprise a sizable population in Shenzhen (accounts for 30% of Shenzhen’s total population), the failure to include parents with other occupations made it hard to generalize our findings to all parents in Shenzhen. Fourth, this study was only conducted in one Chinese city. As compared to other parts of China, Shenzhen is more developed and with better accessibility to SIV and other health-related services. In addition, the pilot scheme under the national SIV program providing free SIV to children has been implemented in Shenzhen. Therefore, the parental hesitancy for SIV among parents in Shenzhen might be lower than less-developed areas in China. Fifth, we were not able to collect information from factory workers who refused to join this study. The participants and refusals might have different characteristics and hesitancy toward SIV. Selection bias existed but is expected to be limited due to the high response rate. Moreover, we constructed some scales to measure perceptions related to SIV in this study. Although the Cronbach’s alpha was acceptable for these scales, they were not validated by separate studies. Furthermore, data were self-reported, and validation was not feasible due to the study design. Parents might under-report hesitancy toward SIV for children due to social desirability. Last but not least, this study was cross-sectional and cannot establish causality.

## 5. Conclusions

Level of parental hesitancy toward SIV for children under the age of 18 remained high in the post-pandemic era. The rebound of seasonal influenza in the post-pandemic era highlights the needs to address parental hesitancy toward SIV for children. Making use of co-creation to improve health promotion materials and modifying perceptions related to seasonal SIV might be useful strategies.

## Figures and Tables

**Table 1:** Background characteristics of participants (n = 1175).

Characteristics	All Participants (n = 1175)	Mothers (n = 526)	Fathers (n = 649)	*p* Value
	N (%)	N (%)	N (%)	
Characteristics of the parents				
Age group, years				
18–25	294 (25.0)	64 (12.2)	230 (35.4)	<0.001
26–30	242 (20.6)	112 (21.3)	130 (20.0)	
31–35	308 (26.2)	176 (33.5)	132 (20.3)	
36–53	331 (28.2)	174 (33.0)	157 (24.3)	
Sex assigned at birth				
Male	649 (55.2)	0 (0.0)	649 (100.0)	N.A.
Female	526 (44.8)	526 (100.0)	0 (0.0)	
Ethnic group				
Han	1101 (93.7)	485 (92.2)	616 (94.9)	0.06
Others	74 (6.3)	41 (7.8)	33 (5.1)	
Permanent residents of Shenzhen				
No	775 (66.0)	387 (73.6)	388 (59.8)	<0.001
Yes	400 (34.0)	139 (26.4)	261 (40.2)	
Education level				
Junior high school or below	174 (14.8)	87 (16.5)	87 (13.4)	0.27
Senior high school or equivalent	256 (21.8)	290 (55.2)	361 (55.6)	
College and above	745 (63.4)	149 (28.3)	201 (31.0)	
Monthly personal income, CNY (USD)				
<5000 (694)	637 (54.2)	334 (63.5)	303 (46.7)	<0.001
5000–6999 (694–972)	394 (33.6)	161 (30.6)	233 (35.9)	
7000–9999 (972–1389)	85 (7.2)	17 (3.2)	68 (10.5)	
>10,000 (1389)	59 (5.0)	14 (2.7)	45 (6.9)	
Type of work				
Frontline workers	783 (66.6)	304 (57.8)	479 (73.8)	<0.001
Management staff	392 (33.4)	222 (42.2)	170 (26.2)	
History of confirmed COVID-19 infection				
No	181 (15.4)	91 (17.3)	90 (13.9)	0.11
Yes	994 (84.6)	435 (82.7)	559 (86.1)	
Confirmed seasonal influenza infection in the past six months				
No	841 (71.6)	412 (78.3)	429 (66.1)	<0.001
Yes	334 (28.4)	114 (21.7)	220 (33.9)	
Other confirmed upper respiratory infections in the past six months				
No	914 (77.8)	444 (84.4)	470 (72.4)	<0.001
Yes	261 (22.2)	82 (15.6)	179 (27.6)	
Number of doses of COVID-19 vaccination received by the participants				
0	70 (6.0)	32 (6.1)	38 (5.9)	0.005
1	98 (8.3)	32 (6.1)	66 (10.2)	
2	251 (21.4)	96 (18.3)	155 (23.9)	
3	703 (59.8)	344 (65.4)	359 (55.3)	
4	39 (3.3)	18 (3.3)	21 (3.2)	
5	14 (1.2)	4 (0.8)	10 (1.5)	
Characteristics of the index child				
Age of index child, years				
<4	252 (21.4)	101 (19.2)	151(23.3)	0.30
4–6	233 (19.8)	110 (20.9)	123 (19.0)	
7–12	520 (44.3)	242 (46.0)	278 (42.8)	
13–17	170 (14.5)	73 (13.9)	97 (14.9)	
History of confirmed COVID-19 infection				
No	324 (27.6)	163 (31.0)	161 (24.8)	0.02
Yes	851 (72.4)	363 (69.0)	488 (75.2)	
Confirmed seasonal influenza infection in the past six months				
No	851 (72.4)	397 (75.5)	454 (70.0)	0.04
Yes	324 (27.6)	129 (24.5)	195 (30.0)	
Other confirmed upper respiratory infections in the past six months				
No	813 (69.2)	386 (73.4)	427 (65.8)	0.005
Yes	362 (30.8)	140 (26.6)	222 (34.2)	

N.A.: not applicable.

**Table 2:** Descriptive statistics of seasonal influenza vaccination hesitancy and independent variables of interest (n = 1175).

	All Participants (n = 1175) (%)	Mothers (n = 526)	Fathers (n = 649)	*p* Value
	N (%)	N (%)	N (%)	
Hesitancy to receive seasonal influenza vaccination (SIV)				
The index children had received SIV for the incoming flu season				
No	776 (66.0)	343 (65.2)	433 (66.7)	0.59
Yes	399 (34.0)	183 (34.8)	216 (33.3)	
Likelihood of vaccinating the index child against seasonal influenza in the next year (among 776 participants whose index child had not received SIV for the incoming flu season)				
Very unlikely/unlikely/neutral	436 (56.2)	168 (49.0)	268 (61.9)	0.008
Likely/very likely	340 (43.8)	175 (51.0)	165 (38.1)	
Parental hesitancy toward SIV for the index child				
No	739 (62.9)	358 (68.1)	381 (58.7)	<0.001
Yes	436 (37.1)	168 (31.9)	268 (41.3)	
Participants had received SIV for the incoming flu season				
No	883 (75.1)	443 (84.2)	440 (67.8)	<0.001
Yes	292 (24.9)	83 (15.8)	209 (32.2)	
Likelihood of receiving SIV in the next year (among 883 participants who had not received SIV for the incoming flu season)				
Very unlikely/unlikely/neutral	566 (64.1)	172 (50.1)	294 (66.8)	0.07
Likely/very likely	317 (35.9)	171 (49.9)	146 (33.2)	
Parents’ hesitancy to receive SIV				
No	609 (51.8)	254 (48.3)	355 (54.7)	0.03
Yes	566 (48.2)	272 (51.7)	294 (45.3)	
Individual-level factors				
Satisfaction of governmental health promotion materials				
Satisfaction of seasonal influenza vaccination health promotional materials (e.g., advertisement, poster, and others) produced by the government, agree				
Can offer the appropriate amount of information related to seasonal influenza vaccination	621 (52.9)	316 (60.1)	305 (47.0)	<0.001
Can address your concerns related to seasonal influenza vaccination	575 (48.9)	251 (47.7)	324 (49.9)	<0.001
Helpful for you to make the decision on whether to receive seasonal influenza vaccination	601 (51.1)	272 (51.7)	329 (50.7)	<0.001
Satisfaction with Health Promotional Materials Scale ^a^, mean (SD)	6.9 (1.8)	7.2 (1.7)	6.7 (1.9)	<0.001
Perceptions related to SIV				
Perceived susceptibility to seasonal influenza for the index children, agree				
The index child has a high chance of contracting seasonal influenza	494 (42.0)	199 (37.8)	295 (45.5)	<0.001
Item score, mean (SD)	2.1 (0.8)	2.1 (0.8)	2.1 (0.9)	0.99
Perceived severity of seasonal influenza for the index child, agree				
The index child will have severe symptoms due to seasonal influenza infection (e.g., persistent cough, fever, pneumonia)	579 (49.3)	243 (46.2)	336 (51.8)	<0.001
Seasonal influenza will have a significant negative impact on your index child’s academic performance	688 (58.6)	312 (59.3)	376 (57.9)	<0.001
Taking care of the index child with seasonal influenza will have a significant negative impact on your work and income	713 (60.7)	323 (61.4)	390 (60.1)	<0.001
Perceived Severity Scale ^b^, mean (SD)	7.1 (2.0)	7.2 (1.8)	7.0 (2.1)	0.03
Perceived benefits of SIV for the index child, agree				
SIV is highly effective in protecting the index child against seasonal influenza	732 (62.3)	325 (61.8)	407 (62.7)	<0.001
SIV is highly effective in reducing the index child’s risk of having severe consequences following seasonal influenza infection	690 (58.7)	317 (60.3)	373 (57.5)	<0.001
Perceived Benefit Scale ^c^, mean (SD)	4.9 (1.4)	5.0 (1.2)	4.8 (1.5)	<0.001
Perceived barriers to have the index child received SIV, agree				
The index child will have severe side effects after receiving SIV	449 (38.2)	178 (33.8)	271 (41.8)	<0.001
Other methods (e.g., mask-wearing) are sufficient to protect the index children against seasonal influenza	723 (61.5)	326 (62.0)	397 (61.2)	<0.001
The location and time to receive SIV are not convenient for you	511 (43.5)	202 (38.4)	309 (47.6)	<0.001
Perceived Barrier Scale ^d^, mean (SD)	6.7 (1.9)	6.7 (1.7)	6.7 (2.0)	0.57
Cue to action related to SIV for the index child, agree				
Your significant others (e.g., doctors, friends, and families) suggested you vaccinate the index child against seasonal influenza	683 (58.1)	310 (58.9)	373 (57.5)	<0.001
Item score, mean (SD)	2.4 (0.8)	2.5 (0.7)	2.3 (0.8)	0.003
Perceived self-efficacy related to SIV for the index child, agree				
It is easy for you to vaccinate the index child against seasonal influenza if you want to	699 (59.9)	326 (62.0)	373 (57.5)	<0.001
Item score, mean (SD)	2.4 (0.8)	2.5 (0.7)	2.3 (0.8)	<0.001
Index child’s use of personal preventive measures				
Index child’s frequency of wearing masks in public transportation in the past month				
Never/not applicable	64 (5.4)	32 (6.1)	32 (4.9)	<0.001
Sometimes	335 (28.5)	190 (36.1)	145 (22.3)	
Always	399 (34.0)	150 (28.5)	249 (38.4)	
Every time	377 (32.1)	154 (29.3)	223 (34.4)	
Item score, mean (SD)	2.9 (0.9)	2.8 (0.9)	3.0 (0.9)	<0.001
Index child’s frequency of wearing masks at schools in the past month				
Never/not applicable	150 (12.8)	88 (16.7)	62 (9.6)	<.001
Sometimes	372 (31.7)	205 (39.0)	167 (25.7)	
Always	353 (30.0)	129 (24.5)	224 (34.5)	
Every time	300 (25.5)	104 (19.8)	196 (30.2)	
Item score, mean (SD)	2.7 (1.0)	2.5 (1.0)	2.9 (1.0)	<0.001
Interpersonal-level factors				
Frequency of exposure to the following information on social media platforms (e.g., WeChat moments, Weibo, Tiktok, Red) in the past month				
Testimonials given by parents about their children’s severe symptoms following seasonal influenza or other upper respiratory infection				
Item score, mean (SD)	1.6 (1.0)	1.6 (1.0)	1.5 (1.0)	0.07
Almost none	235 (20.0)	103 (19.6)	132 (20.3)	
Seldom	267 (22.7)	106 (20.2)	161 (24.8)	
Sometimes	429 (36.5)	194 (36.9)	235 (36.2)	
Always	244 (20.8)	123 (23.4)	121 (18.6)	0.11
Difficulties in seeking medical treatments for influenza or other upper respiratory infections for children				
Item score, mean (SD)	1.6 (1.0)	1.7 (1.0)	1.6 (1.0)	0.005
Almost none	201 (17.1)	89 (16.9)	112 (17.3)	
Seldom	289 (24.6)	108 (20.5)	181 (27.9)	
Sometimes	437 (37.2)	194 (36.9)	243 (37.4)	
Always	248 (21.1)	135 (25.7)	113 (17.4)	0.001
Increasing number of patients in the hospitals due to influenza or other upper respiratory infections				
Item score, mean (SD)	1.8 (1.0)	2.0 (1.0)	1.7 (1.0)	<0.001
Almost none	179 (15.2)	73 (13.9)	106 (16.3)	
Seldom	241 (20.5)	80 (15.2)	161 (24.8)	
Sometimes	404 (34.4)	174 (33.1)	230 (35.4)	
Always	351 (29.9)	199 (37.8)	152 (23.4)	<0.001
Increasing number of severe cases of influenza or other upper respiratory infections				
Item score, mean (SD)	1.7 (1.0)	1.8 (1.0)	1.6 (1.0)	0.004
Almost none	179 (15.2)	75 (14.3)	104 (16.0)	
Seldom	290 (24.7)	114 (21.7)	176 (27.1)	
Sometimes	454 (38.6)	202 (38.4)	252 (38.8)	
Always	252 (21.5)	135 (25.7)	117 (18.0)	0.007
Thoughtful consideration about veracity of information specific to SIV in the past month				
Item score, mean (SD)	1.7 (1.0)	1.8 (1.0)	1.7 (1.0)	0.04
Almost none	180 (15.3)	81 (15.4)	99 (15.3)	
Seldom	260 (22.1)	98 (18.6)	162 (25.0)	
Sometimes	455 (38.7)	205 (39.0)	250 (38.5)	
Always	280 (23.8)	142 (27.0)	138 (21.3)	0.03

^a^ Satisfaction with Health Promotional Materials Scale, 3 items, Cronbach’s alpha: 0.76; one factor was identified via exploratory factor analysis, explaining 60.0% of total variance. ^b^ Perceived Severity Scale, 3 items, Cronbach’s alpha: 0.76; one factor was identified via exploratory factor analysis, explaining 68.1% of total variance. ^c^ Perceived Benefit Scale, 2 items, Cronbach’s alpha: 0.78; one factor was identified via exploratory factor analysis, explaining 82.1% of total variance. ^d^ Perceived Barrier Scale, 3 items, Cronbach’s alpha: 0.67; one factor was identified via exploratory factor analysis, explaining 60.2% of total variance.

**Table 3:** Associations between background characteristics and seasonal influenza vaccination hesitancy among participants.

Characteristics	All Participants (n = 1175)		Mothers (n = 526)		Fathers (n = 649)	
	OR (95%CI)	*p* Value	OR (95%CI)	*p* Value	OR (95%CI)	*p* Value
Characteristics of the parents						
Age group, years						
18–25	Reference		Reference		Reference	
26–30	0.50 (0.35, 0.72)	<0.001	0.62 (0.33, 1.15)	0.13	0.42 (0.27, 0.66)	<0.001
31–35	0.55 (0.39, 0.76)	<0.001	0.39 (0.21, 0.70)	0.002	0.83 (0.54, 1.27)	0.83
36–53	0.53 (0.38 0.73)	<0.001	0.42 (0.23, 0.75)	0.004	0.67 (0.44, 1.01)	0.67
Sex assigned at birth						
Male	Reference		N.A.	N.A.	N.A.	N.A.
Female	0.67 (0.52, 0.85)	<0.001	N.A.		N.A.	
Ethnic group						
Han	Reference		Reference		Reference	
Others	0.86 (0.53, 1.39)	0.53	1.40 (0.73, 2.70)	0.31	1.05 (0.52, 2.13)	0.89
Permanent residents of Shenzhen						
No	Reference		Reference		Reference	
Yes	1.04 (0.81, 1.34)	0.74	0.94 (0.62, 1.43)	0.77	1.01 (0.73, 1.38)	0.97
Education level						
Junior high school or below	Reference		Reference		Reference	
Senior high school or equivalent	1.09 (0.74, 1.61)	0.65	0.89 (0.54, 1.45)	0.63	0.95 (0.59, 1.52)	0.82
College and above	0.70 (0.50, 0.98)	0.04	0.38 (0.21, 0.68)	0.001	0.69 (0.41, 1.15)	0.15
Monthly personal income, CNY (USD)						
<5000 (694)	Reference		Reference		Reference	
5000–6999 (694–972)	0.96 (0.74, 1.24)	0.74	1.10 (0.74, 1.64)	0.64	0.79 (0.58, 1.12)	0.19
7000–9999 (972–1389)	0.81 (0.50, 1.30)	0.38	0.88 (0.30, 2.57)	0.82	0.65 (0.37, 1.12)	0.12
>10,000 (1389)	0.98 (0.56, 1.70)	0.93	0.16 (0.02, 1.26)	0.08	1.10 (0.59, 2.07)	0.76
Type of work						
Frontline workers	Reference		Reference		Reference	
Management staff	0.63 (0.49, 0.82)	<0.001	0.50 (0.34, 0.73)	<0.001	0.87 (0.61, 1.25)	0.45
History of confirmed COVID-19 infection						
No	Reference		Reference		Reference	
Yes	1.18 (0.85, 1.62)	0.33	1.68 (1.06, 2.68)	0.03	0.89 (0.57, 1.41)	0.62
Confirmed seasonal influenza infection in the past six months						
No	Reference		Reference		Reference	
Yes	0.63 (0.48, 0.83)	<0.001	0.71 (0.45, 1.13)	0.15	0.53 (0.38, 0.74)	<0.001
Other confirmed upper respiratory infections in the past six months						
No	Reference		Reference		Reference	
Yes	0.58 (0.43, 0.78)	<0.001	0.60 (0.35, 1.03)	0.07	0.50 (0.35, 0.72)	<0.001
Number of doses of COVID-19 vaccination received by the participants						
0	Reference		Reference		Reference	
1	1.27 (0.68, 2.37)	0.45	1.14 (0.42, 3.12)	0.80	1.29 (0.58, 2.89)	0.53
2	1.10 (0.64, 1.88)	0.74	1.14 (0.50, 2.60)	0.76	1.05 (0.51, 2.15)	0.90
3	0.74 (0.45, 1.23)	0.25	0.64 (0.30, 1.35)	0.24	0.86 (0.44, 1.69)	0.66
4	1.43 (0.65, 3.14)	0.38	1.06 (0.32, 3.48)	0.92	1.83 (0.62, 5.39)	0.27
5	0.83 (0.25, 2.75)	0.77	1.67 (0.21, 13.43)	0.63	0.59 (0.13, 2.64)	0.49
Characteristics of the index child						
Age of index child, years						
<4	Reference		Reference		Reference	
4–6	0.76 (0.52, 1.10)	0.15	0.59 (0.33, 1.07)	0.08	0.93 (0.57, 1.52)	0.78
7–12	0.98 (0.72, 1.34)	0.91	0.77 (0.48, 1.26)	0.30	1.20 (0.80, 1.80)	0.37
13–17	1.01 (0.68, 1.51)	0.95	0.81 (0.43, 1.53)	0.52	1.19 (0.71, 2.00)	0.51
History of confirmed COVID-19 infection						
No	Reference		Reference		Reference	
Yes	0.77 (0.59, 0.99)	0.046	0.53 (0.36, 0.78)	0.001	0.98 (0.68, 1.41)	0.92
Confirmed seasonal influenza infection in the past six months						
No	Reference		Reference		Reference	
Yes	0.74 (0.56, 0.97)	0.03	0.63 (0.40, 0.99)	0.05	0.77 (0.55, 1.09)	0.14
Other confirmed upper respiratory infections in the past six months						
No	Reference		Reference		Reference	
Yes	0.45 (0.34, 0.59)	<0.001	0.42 (0.26, 0.66)	<0.001	0.43 (0.30, 0.61)	<0.001

OR: crude odds ratios; CI: confidence interval; N.A.: not applicable.

**Table 4:** Associations of satisfaction with health promotional materials, perceptions, influence of social media, and behaviors related to mask-wearing with seasonal influenza vaccination hesitancy for the index children.

	All Participants (n = 1175)		Mothers (n = 526)		Fathers (n = 649)	
Factors	AOR (95%CI)	*p* Value	AOR (95%CI)	*p* Value	AOR (95%CI)	*p* Value
Individual-level factors						
Satisfaction of governmental health promotion materials						
Satisfaction with Health Promotional Materials Scale	0.75 (0.69, 0.80)	<0.001	0.69 (0.61, 0.78)	<0.001	0.76 (0.70, 0.84)	<0.001
Perceptions related to SIV						
The index child has a high chance of contracting seasonal influenza (perceived susceptibility)	0.85 (0.73, 0.99)	0.03	0.84 (0.66, 1.08)	0.17	0.86 (0.73, 1.01)	0.06
Perceived Severity Scale	0.83 (0.77, 0.88)	<0.001	0.81 (0.73, 0.90)	<0.001	0.83 (0.76, 0.90)	<0.001
Perceived Benefit Scale	0.67 (0.60, 0.73)	<0.001	0.56 (0.48, 0.66)	<0.001	0.71 (0.63, 0.80)	<0.001
Perceived Barrier Scale	0.93 (0.87, 0.99)	0.02	0.92 (0.82, 1.03)	0.14	0.92 (0.84, 1.00)	0.05
Your significant others (e.g., doctors, friends, and families) suggested you vaccinate the index child against seasonal influenza (cue to action)	0.59 (0.50, 0.69)	<0.001	0.46 (0.35, 0.60)	<0.001	0.64 (0.53, 0.79)	<0.001
It is easy for you to vaccinate the index child against seasonal influenza if you want to (perceived self-efficacy)	0.59 (0.50,0.70)	<0.001	0.50 (0.38, 0.66)	<0.001	0.65 (0.53, 0.79)	<0.001
Index child’s use of personal preventive measures						
Index child’s frequency of wearing masks in public transportation in the past month	0.88 (0.76, 1.01)	0.07	0.85 (0.68, 1.04)	0.12	0.90 (0.74, 1.08)	0.26
Index child’s frequency of wearing masks at schools in the past month	0.91 (0.80, 1.04)	0.15	0.94 (0.77, 1.14)	0.50	0.87 (0.74, 1.04)	0.12
Parents’ hesitancy to receive SIV						
No	Reference		Reference		Reference	
Yes	4.46 (3.38, 5.90)	<0.001	5.22 (3.35, 8.14)	<0.001	3.91 (2.74, 5.58)	<0.001
Interpersonal-level factors						
Frequency of exposure to the following information on social media platforms (e.g., WeChat moments, Weibo, Tiktok, Red) in the past month						
Testimonials given by parents about their children’s severe symptoms following seasonal influenza or other upper respiratory infection	0.85 (0.76, 0.96)	0.009	0.86 (0.71, 1.03)	0.11	0.86 (0.73, 1.01)	0.06
Difficulties in seeking medical treatments for influenza or other upper respiratory infections for children	0.87 (0.77, 0.99)	0.03	0.92 (0.76, 1.11)	0.40	0.86 (0.73, 1.01)	0.07
Increasing number of patients in the hospitals due to influenza or other upper respiratory infections	0.85 (0.75, 0.96)	0.008	0.89 (0.74, 1.07)	0.22	0.84 (0.71, 0.98)	0.03
Increasing number of severe cases of influenza or other upper respiratory infections	0.88 (0.77, 0.99)	0.04	1.01 (0.83, 1.23)	0.91	0.79 (0.67, 0.94)	0.008
Thoughtful consideration about veracity of information specific to SIV in the past month	0.93 (0.82, 1.05)	0.23	1.01 (0.83, 1.23)	0.94	0.89 (0.76, 1.05)	0.17

AOR: adjusted odds ratios, odds ratios adjusted for significant background characteristics listed in Table 3; CI: confidence interval.

## Data Availability

The datasets generated and/or analyzed during the current study are not publicly available as they contain sensitive personal information but are available from the corresponding author upon reasonable request.

## Appendix A

**Table A1:** A correlation matrix between information exposure on social media and perceived susceptibility and perceived severity among participants (n = 1175).

		Perceived Susceptibility		Perceived Severity Scale	
Information Exposure on Social Media		OR (95%CI)	*p* Value	OR (95%CI)	*p* Value
Frequency of exposure to the following information on social media platforms (e.g., WeChat moments, Weibo, Tiktok, Red) in the past month					
	Testimonials given by parents about their children’s severe symptoms following seasonal influenza or other upper respiratory infection	2.20 (2.11, 2.83)	<0.001	7.30 (7.09, 7.50)	<0.001
	Difficulties in seeking medical treatments for influenza or other upper respiratory infections for children	2.17 (2.08, 2.26)	<0.001	7.12 (6.90, 7.33)	<0.001
	Increasing number of patients in the hospitals due to influenza or other upper respiratory infections	2.26 (2.17, 2.35)	<0.001	7.19 (7.00, 7.42)	<0.001
	Increasing number of severe cases of influenza or other upper respiratory infections	2.22 (2.13, 2.31)	<0.001	7.31 (7.09, 7.53)	<0.001

OR: crude odds ratios; CI: confidence interval.
